# A Gelated Colloidal Crystal Attached Lens for Noninvasive Continuous Monitoring of Tear Glucose

**DOI:** 10.3390/polym9040125

**Published:** 2017-03-28

**Authors:** Jia-Li Ruan, Cheng Chen, Jian-Hua Shen, Xue-Ling Zhao, Shao-Hong Qian, Zhi-Gang Zhu

**Affiliations:** 1 Department of Ophthalmology, EENT Hospital of Fudan University, Shanghai 200031, China; jruan1203@hotmail.com; 2 School of Environmental and Materials Engineering, College of Engineering, Shanghai Polytechnic University, Shanghai 201209, China; chencheng@sspu.edu.cn (C.C.); xlzhao@sspu.edu.cn (X.-L.Z.); 3 Key Laboratory for Ultrafine Materials of Ministry of Education, School of Materials Science and Engineering, East China University of Science and Technology, Shanghai 200237, China; jianhuashen@ecust.edu.cn

**Keywords:** tear glucose, photonic crystal, contact lens, cytotoxicity, hydrogel, diffraction

## Abstract

Patients of diabetes mellitus urgently need noninvasive and continuous glucose monitoring in daily point-of-care. As the tear glucose concentration has a positive correlation with that in blood, the hydrogel colloidal crystal integrated into contact lens possesses promising potential for noninvasive monitoring of glucose in tears. This paper presents a new glucose-responsive sensor, which consists a crystalline colloidal array (CCA) embedded in hydrogel matrix, attached onto a rigid gas permeable (RGP) contact lens. This novel sensing lens is able to selectively diffract visible light, whose wavelength shifts between 567 and 468 nm according to the alternation of the glucose concentration between 0 and 50 mM and its visible color change between reddish yellow, green, and blue. The detection limit of responsive glucose concentration can be reduced to 0.05 mM. Its combination with a contact lens endows it with excellent biocompatibility and portability, which shows great possibility for it to push the development of glucose-detecting devices into new era.

## 1. Introduction

The desire for noninvasive and continuous monitoring glucose in the human body has increased in importance due to the concern associated with the increasing incidence of diabetes worldwide [1,2,3]. Lots of attention has been paid to the easily accessible body fluids like urine [4,5,6,7], sweat [8,9,10], and especially tears [11,12,13,14,15], which have been confirmed to participate in glucose metabolism and have a positive correlation with the alternation of the concentration of blood glucose [16,17,18,19]. The ideal sensing technology would be portable, inexpensive, and able to selectively and sensitively detect glucose with painless touch and real-time feedback.

The typical glucose range in tear fluid is around 0.1–0.6 mM [20]. Though having a promising potential for glucose monitoring in the human body due to its correlation with blood glucose, the tear glucose sensors are not like traditional implanted glucose biosensors, due to their specific characteristics: limited amount fluid, low generation rate, low glucose concentration, rich chemical composition, etc. Thus, numerous methods have been used to detect glucose in tears, including sophisticated analytical techniques such as electrochemistry [21,22,23,24,25,26], chromatography [14], mass spectrometry [27], fluorescence [28,29], Raman spectrometry [30], and many others. However, these approaches face disadvantages of utilizing precise instruments and requiring highly trained personnel.

Asher’s group firstly introduced polymerized crystalline colloidal array (PCCA) sensors into glucose detecting area [12,31,32,33], which have great possibility to be developed into point-of-care device since they can offer fast and visual detection of analytes through colorimetric determinations of concentration. Crystalline colloidal arrays (CCAs) generally possess three-dimensional (3D) periodic face-centered cubic (FCC) lattice that self-assembled from monodisperse colloidal spheres [31]. The highly ordered structure of CCA can be permanently locked in a hydrogel matrix by polymerization of a monomer around the CCA spheres to form a PCCA [34]. Three primary mechanisms of PCCA sensing have been summarized including the change of the hydrogel crosslink density, immobilization of ions into the hydrogel, and the change in free energy after mixing the hydrogel polymer with the aqueous medium. The accompanying result is that the polymer matrix either swells or shrinks, leading to red- or blue-shift of the Bragg diffraction and in turn their color will change [31,35]. Although this is a simple and cheap method, it also suffers from low sensitivity and slow response time.

Lenses, usually used for vision correction as a portable and accessible device [36], with their favorable biocompatibility with decades of clinical use, now have caught much attention in drug delivery and tear analytes detection fields [37,38]. A glucose-sensitive contact lens was prepared by immobilizing two types of fluorescent indicators in the lens material as it is polymerized [39]. In the presence of glucose, the indicators dissociated and the fluorescence was detected. The signal was read with the aid of an illumination/recording unit held in front of the eye. Another method was constructing an electrochemical sensor on lens [40]. This approach showed enough sensitivity for tear glucose, however it was unknown what role the interfering electroactive species present in tear fluid played.

In this paper, we designed a new sensor device by embedding a three-dimensional polystyrene (PS) CCA in 4-boronobenzaldehyde (4-BBA)-modified poly(vinyl alcohol) (PVA) hydrogel, which was attached onto a rigid gas permeable contact lens, thus forming a hydrogel-based CCA-lens to monitor tear glucose. Such physical gelation method enables the construction of hydrogel-based CCA on the irregular surface. Due to its dielectric periodicity, this material is able to selectively diffract electromagnetic waves of certain frequencies according to Bragg’s law. Thanks to the diols and borate ions combining with glucose to alter the hydrogel volume, the PS particle space changes automatically and thus leads to the shift of the diffraction wavelength, seen as structure color offset.

## 2. Material and Methods

### 2.1. Materials

All materials were used as received unless otherwise specified. d-(+)-Glucose (99.5%) was purchased from Sigma-Aldrich (St. Louis, MO, USA). Ultrapure water (18.2 MΩ·cm) was obtained from synergy U.V. Millipore water purification system. Rigid gas permeable (RGP) contact lenses (0.00 in luminosity, 7.3 in base curve and 11.0 mm in diameter) polymerized from polymethyl methacrylate (PMMA) were obtained from Alcon, Shanghai, China. PVA (99% hydrolyzed, DP = 1750 ± 50) was purchased from Shanghai Chemical Agent Co., Ltd., Shanghai, China. Human corneal epithelial cells (HCECs) were provided by Dr. Xu of EENT hospital of Fudan University (Shanghai, China). Fetal bovine serum (FBS) without mycoplasma, penicillin–streptomycin double antibiotics, and Dulbecco’s Modified Eagle Medium (DMEM) were purchased from Gibco, Grand Island, NY, USA. Calcein acetoxymethyl (AM) ester and propidium iodide (PI) was purchased from Molecular Probes, Eugene, OR, USA. CCK-8 Proliferation Assay kits and albumin were purchased from Sigma Aldrich. All other reagents were of analytical grade and obtained from Sigma-Aldrich.

### 2.2. Self-Assembly of PS Colloids on Contact Lens

The monodisperse PS with diameter of ~200 nm was prepared as reported before [41]. The PS particles were self-assembled by vertical sedimentation methods on RGP contact lenses. The lenses were first hydrophilization processed by ultrasonic cleaning with deionized water after soaking in H_2_O_2_ (5 wt %) solution for 12 h and dried. Coverslips were ultrasonically cleaned and dried using acetone, ethanol, and deionized water for 15 min after immersion in concentrated H_2_SO_4_-H_2_O_2_ (7:3, *V*/*V*) solution for 6 h. The contact lens was fixed on the surface of coverslips before vertically affixed to the glass tank, then 0.3 wt % PS microspheres suspension was added. At the constant temperature of 60 °C the solution uniform evaporated, leading PS particles to self-assemble into colloidal crystal on the surface of the contact lens by its surface tension.

### 2.3. Gelation of CCA-Lens by Glucose-Responsive PVA

**Figure 1:**
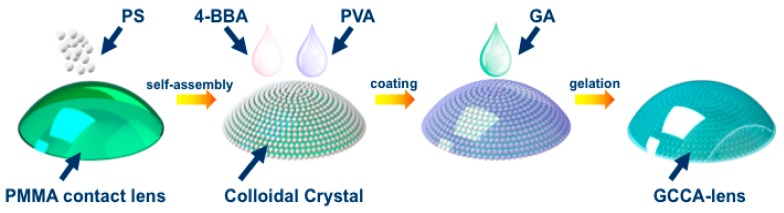
The preparation route of the 4-BBA-PVA GCCA-lens.

For the purpose of glucose detection, PVA solution was modified with a typical recipe. PVA powder was constantly stirred in dimethyl sulfoxide (DMSO) in the atmosphere of N_2_ and heating-up temperature of 120 °C for 2 h, then 0.4 g of 4-BBA and drops of HCl were added for reaction. After being cooled down, 10 wt % 4-BBA-PVA homogeneous solution was prepared, with which the CCA-lens mentioned above was coated for 5 h’s standing to form physical gel. Then 1.5 mL 10% glutaraldehyde was added as cross-linking agent and the pH of the system was adjusted to 1 by concentrated sulfuric acid dropwise, the reaction lasted for 4 h with slight stirring. The resultant glucose-detective gelated CCA-lens (GCCA) was rinsed with ultrapure water to end cross-linking. In order to systematically present the construction of the glucose-responsive GCCA-lens, the process is described in the Figure 1.

### 2.4. Cell Culture and Cytotoxicity Test

HCECs were cultured and grown at 37 °C and 5% CO_2_ in sterile DMEM medium with 10 wt % fetal bovine serum and 1 wt % glutamine and penicillin-streptomycin. HCECs were seeded at a density of 1 × 10^4^ cells per well in 96-well tissue culture plate (TCP). According to ISO 10993, CCA-lens extracted in 50% to 200% concentration to culture HCECs for 6, 24, and 48 h, its cytotoxicity was valued by CCK-8 proliferation assay and a scientific microplate reader (MK3, Thermo, Waltham, MA, USA) to determine the optical density (OD) value at 570 µm. Inverted fluorescence microscopy (IFM, Leica, Solms, Germany) observation was employed to evaluate the viability and morphology of HCECs after adding AM and PI for the staining of living cells and dead cells, respectively, after 48 h whose quantification was conducted by Image J (National Institutes of Health, Bethesda, MD, USA).

### 2.5. Characterizations of the CCA Embedded Hydrogel

Pictures were captured to observe the morphology of CCA assembled from PS nanoparticles by scanning electron microscope (SEM, JEOL Ltd., Tokyo, Japan). To test the swelling property of 4-BBA functionalized PVA hydrogel, the sample was immersed in different glucose solution, and the weight change was measured compared with pure PVA. Moreover, by adjusting pH of the solution, their weight alternation was also valued.

### 2.6. Reflection Measurement of the GCCA-Lens

In an in vitro test, to study the glucose-responsive optical properties, glucose was dissolved in both ultrapure water and simulated tear fluid (STF, 6.78 g/L NaCl, 2.18 g/L NaHCO_3_, 1.38 g/L KCl, 0.084 g/L CaCl_2_·2H_2_O, 3.94 g/L albumin, pH 7.4) in concentration range from 0 to 50 mM to simulate human inner environment. After the GCCA-lens was immersed in the glucose solution, the diffraction wavelength was monitored until it was stabilized by a fiber optic spectrometer (Ocean Optics USB 4000-XR1-ES, Ocean Optics, Winter Park, FL, USA). The peak wavelength was then recorded as the color of the lens. It should be noted that, for processing of the data, a background spectra taken of a swollen but colorless film was subtracted to achieve a better signal-to-noise ratio.

### 2.7. Statistical Analysis

Data in this paper was presented as mean ± standard error of the mean of *n* experiments. The statistical analysis involving multiple comparisons was conducted with a one-way analysis of variance (ANOVA) test and two-way ANOVA, respectively. A *p* value of less than 0.05 was considered as significant. GraphPad Prism (v 7, GraphPad Software, San Diego, CA, USA) was used to perform all the statistical calculations.

## 3. Results and Discussion

### 3.1. Biocompatibility Characterization of GCCA-Lens

**Figure 2:**
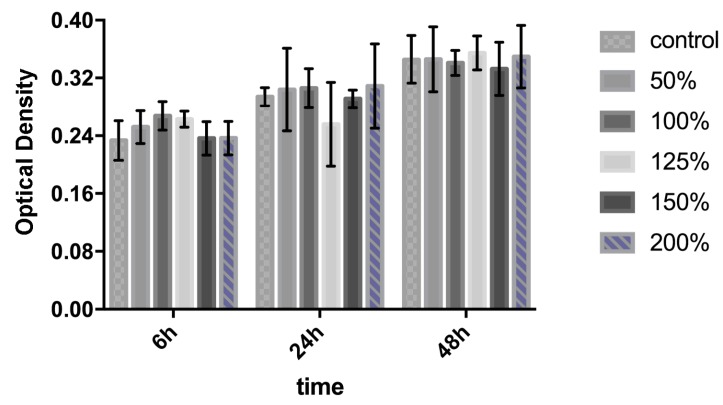
The cytotoxicity of extraction of GCCA-lens in HCECs: CCK-8 assay of the attachment and proliferation viability (*p* > 0.05 vs. control, *n* = 5).

**Figure 3:**
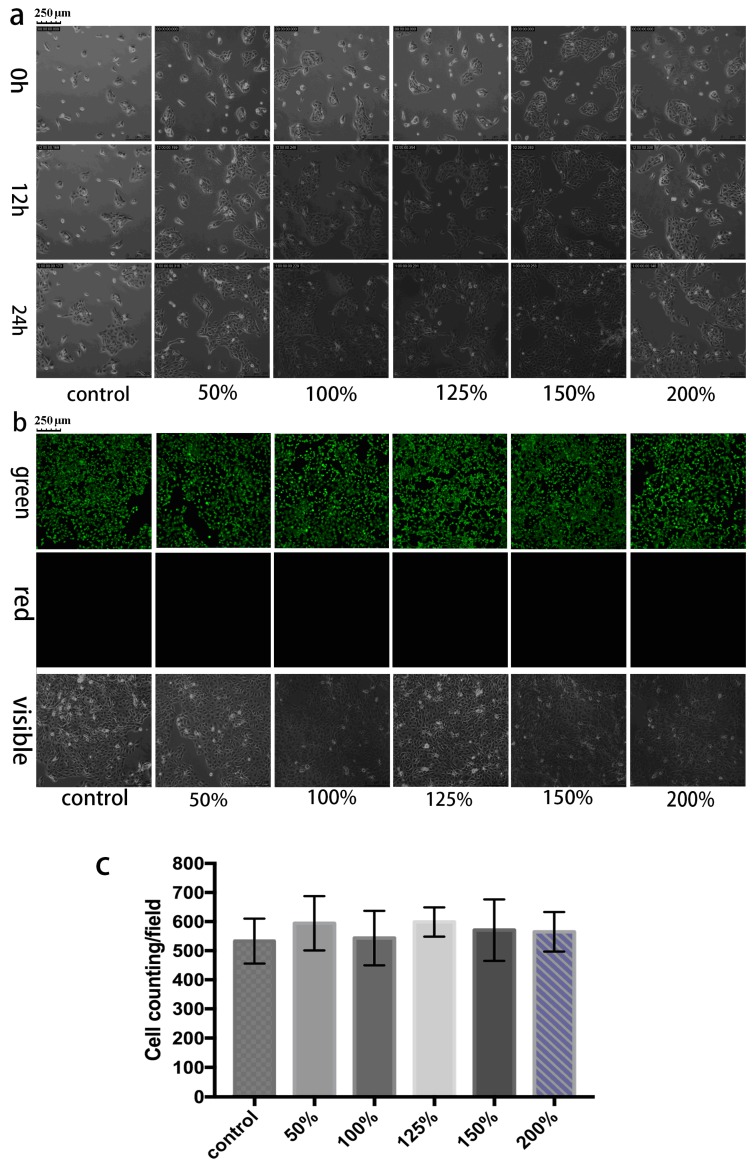
IFM micrographs of HCECs cultured with varied concentration of extraction of GCCA-lens. (**a**) Cells’ growth morphology and migration path in 0–24 h, and their condition in 48 h was shown below in: (**b**) Green (AM) and red (PI) fluorescence micrographs and cells’ visible morphology in 48 h; (**c**) living cell statistical analysis (*p* > 0.05 vs. control, *n* = 3). Scale bars of 250 μm were added for easier reading.

The initial studies verified the use of the sensitive hydrogel-based colloidal crystal as a contact lens sensor. For tear glucose monitoring, the potential toxicity should be taken into careful consideration as the GCCA-lenses are designed for close interaction with the surface of eyes and its surrounding tissue [42,43,44]. Though the sensor was constructed without any acute cytotoxic material, the biocompatibility was firstly examined by quantitative analysis of cell proliferation activity by CCK-8 assay and analysis of morphology from fluorescence micrographs. The final OD values in direct proportion to various concentration of GCCA-lens extraction cultured from 6, 24, 48 h are shown in Figure 2. In control group, the OD value rose up in a moderate rate, while the experiment group showed a similar proliferative rate from 6 to 48 h, which had no statistical difference (*p* > 0.05), suggesting the GCCA-lens has no obvious cytotoxicity to HCEC. HCECs have spindle morphology and polarity with a long axis direction. The morphology of cells in the experiment group stayed the same as the control group illustrated in Figure 3a. After 48 h, AM (green fluorescence) and PI (red fluorescence) staining was captured by IFM presented in Figure 3b, showing similar cell proliferation rate. The living cells were quantified and statistically analyzed, which has no significant difference (*p* > 0.05) in Figure 3c, and no obvious dead cells were found with negative PI staining. This indicates that the as-prepared sensor devices have excellent cytocompatibility to promote cell proliferation.

### 3.2. Swelling Ability of Hydrogel and Sensing Mechanism of GCCA-Lens

**Figure 4:**
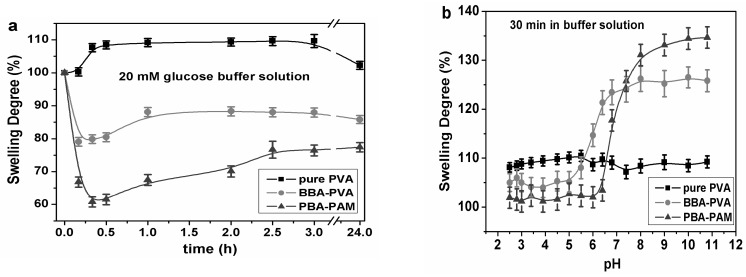
Swelling curves of PVA hydrogel, 4-BBA-PVA and PBA-PAM. (**a**) In 20 mM glucose solution; (**b**) In buffered media.

PVA is the product of free radical polymerization of vinyl acetate followed by hydrolysis of acetate groups to hydroxyl moieties [45]. The molecular weight distribution is an important factor in our test, due to its role in determining polymer properties. One crosslinking 4-BBA-PVA and another fluorophenylboronic acid modified polyacrylamide (PBA-PAM) hydrogel [33] were carried out to compare their swelling ability in 20 mM glucose and varied pH condition. As shown in Figure 4a, under 20 mM glucose solution, pure PVA hydrogel swelled 10% while 4-BBA-PVA and PBA-PAM shrunk by 20% and 40% of weight, respectively. Such properties ensured the GCCA-lens of signal magnification of analyte detection.

**Figure 5:**
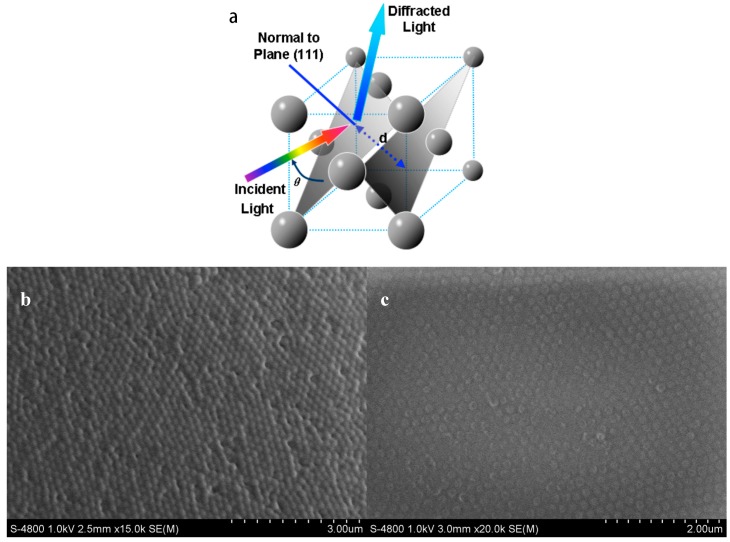
(**a**) Schematic diagram of GCCA’s diffraction phenomenon from the (111) planes of the crystalline colloidal array (CCA) with a FCC arrangement that follows Bragg’s law; (**b**) The SEM photographs of the colloidal crystal assembled from PS nanoshperes; (**c**) 4-BBA-modified PVA hydrogel coated colloidal crystal, the periodic arrangement was successfully embedded in the hydrogel matrix.

We designed the boronic acid functionalized PVA hydrogel as glucose sensitive matrix. The response is due to the acidic nature of boronic acid [46]. Exposure to sugars, like glucose, changes the chemistry of each boronic acid moiety. Boronic acid can generate protons by abstracting a hydroxide unite from water. We examined the swelling property of the above-mentioned hydrogels at different buffer pH for 30 min, as can be seen in Figure 4b, pure PVA hydrogel kept coherent weight while 4-BBA-PVA and PBA-PAM slightly swelled in acidic medium and dramatically swelled in alkali medium at a similar ratio, the pH dependence demonstrated the successful functionalization of PVA by 4-BBA. To support the characterization of the functionalized PVA, the surfaces were examined by Fourier transform infrared spectroscopy (FTIR, Bruker Vertez-70, Bruker, Karlsruhe, Germany) (see the Figure S1 in the Supplementary Materials). We originally expected that the 4-BBA-PVA would show a p*K*a of 7.4 which would give rise to the pH dependence because of the existence of borate. The result showed an effective p*K*a decreased to ~6.0 as the hydrogel reversibly titrated in different buffer medium. Diols such as glucose bind to boronic acid and can decrease the p*K*a of acid, which also increase the negatively charged units. Thus by the change of free energy of mixing and the elastic restoring force, a relationship between changes in osmotic pressure and changes in volume can be developed as long as the system is stable. The change in volume, which is directly proportional to the change in thickness, can explain the shifts in diffracted wavelength by Bragg’s law:

λ = 2*nd*sinθ, (1)

where *d* is thickness of a given layer in this case, λ is wavelength, *n* is refractive index, and θ is the Bragg angle (during measurements, θ = 90°). The diffraction wavelengths result from the Bragg stack nanostructure of the PS CCA and 4-BBA modified PVA, whose interval was called the photonic band gap, a periodic permutation that is an integer multiple of wavelengths of visible light (illustrated in Figure 5a).

As shown in Figure 5, the PS colloidal crystal was well assembled on the surface of the RGP lens forming a close-packed structure (Figure 5b). After coating of the 4-BBA-modified PVA hydrogel, the assembly was embedded in the hydrogel and could be swollen due to the formation of the hydrogel, and thus the crystal array appeared to have non-close-packed morphology (Figure 5c). Hence, with its dielectric periodicity, the diffracted wavelengths are proportional to the volume change of GCCA, the wavelength shifts can thereby be correlated to glucose concentration for the purpose of detection.

### 3.3. Glucose Sensing in Glucose Solution and STF

**Figure 6:**
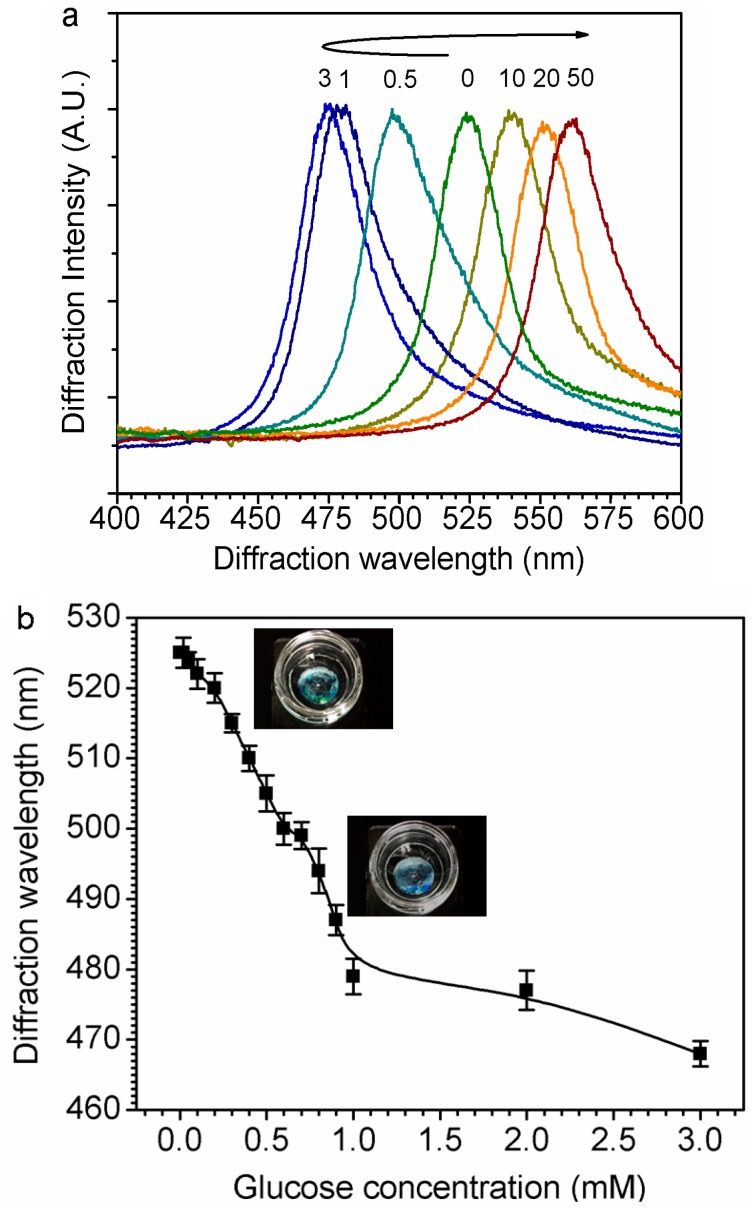
Diffraction wavelength of GCCA-lens shifted responsive to glucose concentration changing. (**a**) Visible color shift of GCCA-lens according to glucose concentration change; (**b**) The diffraction response at low glucose concentration (insert is the photograph of the GCCA-lens sample).

The diffracted color of the GCCA sensor blue-shifted from green to blue, and then red-shifted from blue through green to reddish yellow after it was moved from pure water to gradient glucose solution. Figure 6a indicates the shift of diffraction wavelength after changing glucose concentration from 0 to 50 mM. Specifically, at low glucose concentration, increasing from 0 to 3 mM, the diffraction wavelength shifted from 525 to 468 nm, and then shifted to 567 nm. It had a turning point at 3 mM glucose, before which the diffraction wavelength blue-shifted with the glucose concentration going up and after which it red-shifted otherwise. Since the glucose concentration under physiological conditions is 0.1~0.6 mM, and typical, mean value is 0.16 mM in non-diabetes, the diffraction wavelength shift is capable of going one way without an overlap region and the higher range of glucose concentration will not be discussed here as it does not fit in ordinary physical conditions.

Figure 6b states the diffraction wavelength shift at a relatively lower glucose concentration before the turning point. In the range from 0 to 1 mM covering tear glucose concentration, there is an approximate linear correlation between glucose concentration and diffraction wavelength, mathematically its linear correlation curve can be fitted as:

λ = 526.30885 − 41.83394*C*_glucose_, (2)

where *C*_glucose_ is the concentration of the glucose in buffer solution and λ is the related diffraction wavelength (*R* = 0.99306, *p* < 0.0001).

**Figure 7:**
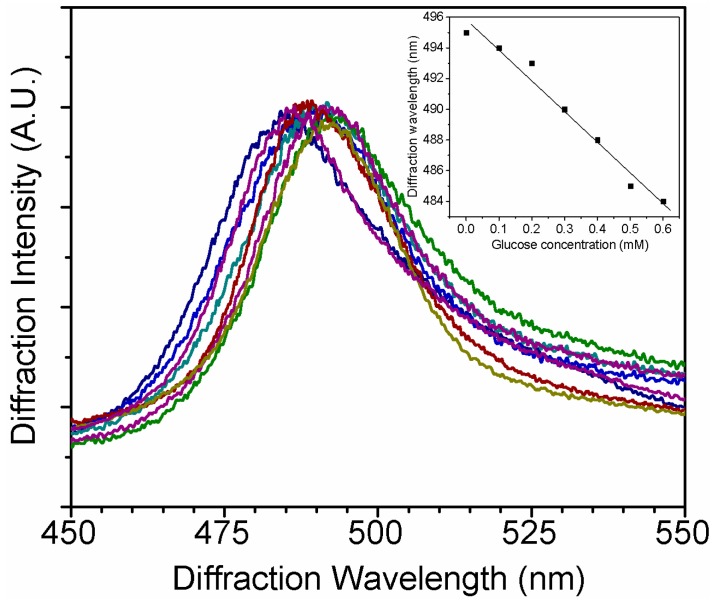
Diffraction wavelength shift under physiological tear glucose concentration (0.1–0.6 mM). Insert is the correlation curve between glucose concentration and diffraction wavelength.

To simulate the sensing performance in tear fluid, different amounts of glucose were dissolved in STF to form a 0.1–0.6 mM solution at a pH of 7.4. The GCCA-Lens sensor demonstrates a 10 nm shift of diffraction wavelength, as shown in Figure 7, which is around one-third of the shift in PBS solution, and the interference might be due to the proteins in STF. It clearly indicates that this sensor is able to detect glucose physiologically in tear fluid, and also has a relative linear correlation between glucose concentration and diffraction wavelength, as shown in the insert of Figure 7. More efforts will be carried out to improve the sensitivity of such sensors in simulated tear fluid.

Compared with the former PCCA constructing way—i.e., UV polymerization, requiring a particular mold—the GCCA-lens structured in this paper was initially constructed by PVA gelation, a first introduced physical way not only for good biocompatibility but also for its capacity of forming gelation on an irregular surface [47,48,49]. The initial color of the GCCA-lens was green, and it shifted to turquoise at 1 mM glucose concentration then to reddish yellow at glucose concentrations higher than 20 mM. This not only allowed differentiation between concentrations of glucose but presented a method to qualitatively determine whether glucose is present from a simple, intense change in color.

## 4. Conclusions

This study constructed a PS colloidal crystal in 4-aldehydephenylboric acid-modified PVA hydrogel, attached onto a rigid gas permeable contact lens, which allowed a simple fabrication, and demonstrates the possibility of detection of glucose. Such GCCA-lens technology conquers the limitations of the PCCAs, and the initial results exhibit the sensor lens’ capability to shift between 567 nm and 468 nm in response to glucose concentrations ranging between 0 and 50 mM. It is noteworthy that the GCCA-lens’ response the color changes between green and blue within the tear glucose concentration region, and is highly visible with exposure to 50 mM glucose invoking a change from green to yellow. Beyond that, the sensor could selectively bind glucose in the presence of other analytes in STF, causing a weak response of ~10 nm. Such properties give its further application for point-of-care, producing a quick and easy method to monitor glucose in tear fluid. Efforts are in progress to make the GCCA-lens sensor more sensitive in STF.

## Supplementary Materials

The following are available online at www.mdpi.com/2073-4360/9/4/125/s1, Figure S1: FTIR spectra of pure PVA and 4-BBA modified PVA samples.
