# Supplementary material for: A Gelated Colloidal Crystal Attached Lens for Noninvasive Continuous Monitoring of Tear Glucose
Source: Polymers (Basel). 2017 Mar 28;9(4):125. doi: 10.3390/polym9040125 (PMC6432312; doi:10.3390/polym9040125)
Supplement: Supplementary file 1 [file polymers-09-00125-s001.pdf]

Supporting Information: A Gelated Colloidal Crystal Attached Lens for Noninvasive Continuous Monitoring of Tear Glucose

Jia-Li Ruan[1†], Cheng Chen[2†], Jian-Hua Shen[3], Xue-Ling Zhao[2], Shao-Hong Qian[1*], and Zhi-Gang Zhu[2*]

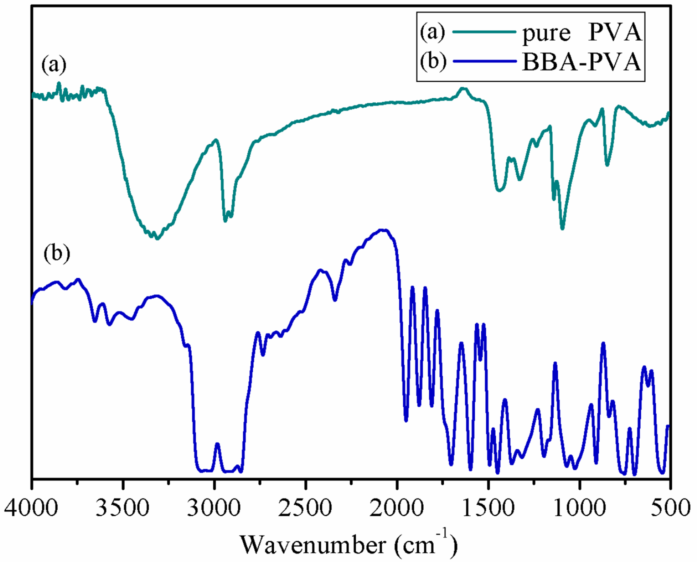

Figure S1. Fourier transform infrared spectroscopy (FTIR) spectra of (**a**) PVA hydrogel and (**b**) 4-BBA-PVA hydrogel.

The surface chemistry of the freshly synthesized hydrogel samples was analyzed by using an FTIR spectrometer (Bruker Vertez-70). Each spectrum was measured in a range between 500 and 4000 cm^{-1}. The result showed peaks at 1450 cm^{-1}, 2950 cm^{-1} and 3300 cm^{-1} correspond to the C-H bending (alkane, $-CH_3$, $-CH_2$) and the C-H stretching vibration from pure PVA, respectively. After the modification of 4-BBA, the absorption peak at ~2900 cm^{-1} was broadened, which belongs to the stretching vibration of -OH group. A =CH stretching vibration appeared around 3100 cm^{-1} demonstrated benzene ring was contained and a C=O stretching vibration appeared about 1700 cm^{-1} showed the modified material structure contained carbonyl. Vibration peaks of benzene derivatives at 2000 cm^{-1} certified that, after the modification, the benzene derivatives—phenylboronic acid in our case,—were grafted onto the PVA chain.
